# A cross-sectional national survey assessing self-reported drug intake behavior, contact with the primary sector and drug treatment among service users of Danish drug consumption rooms

**DOI:** 10.1186/s12954-016-0115-0

**Published:** 2016-10-07

**Authors:** Eva Charlotte Toth, Jette Tegner, Sigurd Lauridsen, Nanna Kappel

**Affiliations:** 1 Department of Nursing, Metropolitan University College, Copenhagen, Denmark; 2 Cowi, Lyngby, Denmark

**Keywords:** Drug consumption room, Harm reduction, Smoking facility

## Abstract

**Background:**

Drug consumption rooms (DCRs) have been implemented worldwide as a harm-reducing strategy. In 2012, Denmark passed legislation allowing establishment of DCRs. The aim of this study was to identify characteristics and gain knowledge of the way service users use the DCRs including bridge building to specialized health care. Associations between nationality, opioid substitution treatment (OST), drug intake method, and response to staff advice on harm-reducing education was investigated, as well as service user’s reasons for using the DCRs, and their perceptions of safety and trust in the DCRs.

**Methods:**

A survey questionnaire sampled 154 participants of DCRs. Convenience sampling was used. Key variables covered demographics, drug intake mode, educational advice received in the DCR, and opinions about and role of the DCRs for the service users.

**Results:**

Only 10 % of the participants were under the age of 30, 30 % between 30 and 39 years, 36 % between 40 and 49 years, and 24 % age 50 or more. A total of 60 % of the participants had encountered drugs before they were 19 years old. Female participants were 25 %, and 73 % were Danish citizens, 8 % were non-Danish EU citizens, and 18 % were non-EU citizens. As drug intake method, 63 % injected drugs in a vein, 7 % sniffed, and 37 % smoked. Of drugs used in the DCR, 49 % used cocaine, 41 % heroin, 16 % a mix of heroin and cocaine, and 16 % used methadone. Participants who smoked drugs made significantly less use of drug rehabilitation than participants who sniffed or injected drugs. There was a similar rate of advice on OST across nationality. Participants accepted staff education on hygienic measures and safe injection practices and found it useful. Participants felt safe and trusted staff and bridge building to specialized health care took place in the DCR.

**Conclusions:**

Staff of Danish DCRs educate service users on health related issues and harm-reducing interventions. A subgroup who smoke and a subgroup of nationality other than Danish are underserved and have less likely been in OST. More research on these groups is needed.

**Electronic supplementary material:**

The online version of this article (doi:10.1186/s12954-016-0115-0) contains supplementary material, which is available to authorized users.

## Background

Illicit drug use is associated with a range of social and environmental harms. The lack of safe environments in which to consume drugs poses a high risk to people who use drugs (PWUD) and drug intake often takes place in public spaces [1–3]. Public drug use contributes to unhygienic injection techniques, causing tissue damages and infections [4, 5], and has negative consequences on the health of PWUD. In major Danish cities, drugs are frequently consumed under stressful and unsafe conditions in public spaces that serve as open drug scenes. PWUD feel ashamed of using drugs in public areas and the exposure of public injecting damages the reputations of individual PWUD [6, 7]. Around open drug scenes, public opinion calls for urban renewal, tidy surroundings, and clean playgrounds. Citizens around open drug scenes, including families with children, have expressed concern over public injecting in the local surroundings [8]. Over the previous 20 years, the number of people in Denmark who die due to drug overdose fluctuates somewhat but averages about 250 people per year [9, 10].

To reduce harm and prevent overdose caused by unsafe drug consumption, various harm reduction policies have been implemented by the Danish Ministry of Health, such as needle exchange programs, free drug treatment and opioid substitution treatment (OST) [10]. By establishing low threshold facilities to reach the vulnerable and socially marginalized PWUD, it is possible to offer health care and build bridges that lead to more specialized health care, OST, and drug-free treatment [6].

One such harm reduction policy is the establishment of supervised drug consumption rooms (DCRs), which have been established worldwide to reducing the harm to which PWUD are exposed [11–13]. A DCR is defined as a “professionally supervised health care facility where drug users can use drugs in safer and more hygienic conditions” [10] than street-based drug using. Over the past 25 years, DCRs have been implemented in several European countries [12] as well as in Australia and Canada [10]. Internationally, the effectiveness of DCRs in harm reduction has been supported by several studies. DCRs attract people with chronic use of drugs, who have increased health risk [14], contribute toward reducing the transmission of infectious diseases such as HIV and hepatitis C, and offer the treatment of infectious wounds [13–20].

In 2012, Denmark passed legislation allowing municipalities to establish DCRs. The bill amending the law presumes that “… within the vicinity of the DCRs, police will not prosecute people who are over the age of 18 years, who possess drugs for their own use, and who have a prolonged use of and addiction to illicit drugs” [7]. The motivation behind this political decision was threefold: to reduce the number of deaths by overdose; to improve life options for PWUD by building bridges to the health care system, drug treatment facilities, and social services; and to reduce drug use related nuisance to surrounding neighborhoods [7]. In the 3 years following this landmark decision, the three largest cities in Denmark have established DCRs. DCRs are organized as integrated, specialized, or mobile models [10, 12]. Currently, more than 3600 service users are registered in Danish drug consumption rooms. Danish DCRs mainly provide service to highly marginalized PWUD, in many cases including OST clients with patterns of stimulant use.

Few studies to date have examined the Danish DCRs. One study of one of the Copenhagen DCRs shows that service users adopt safer behaviors that reduce harm and benefit health [21] and a recent evaluation of the DCRs finds a positive effect on overdose prevention. Other than these, no national study has been performed identifying characteristics of this population and the mode of drug use in the DCRs, nor in assessing the service users’ utilization of the harm reductive interventions delivered in the DCR.

For this reason, this national research study that covers all of the existing DCRs in Denmark was initiated in December 2013. The aim of this article is to identify characteristics of the service user population and gain knowledge of the way service users use the DCRs, including bridge building to specialized health care. Further, we investigate associations between nationality, OST, drug intake method, and response to staff advice on harm-reducing education. Finally, we investigate service users’ reasons for using the DCRs, their perceptions of safety and trust in the DCRs.

## Methods

This paper reports a subset of results from a larger mixed method study of Danish DCRs [22]. Based on reports and files from the five DCRs in Denmark that assess the number of service users, types of drugs, and routes of administration [23, 24], a mixed method study was designed using an explanatory sequential design [22, 25].

The first part of the mixed method study comprised of 250 h of participant observation performed in the DCRs in Denmark. The second part comprised of semi-structured interviews with 25 staff and 40 service users of the DCRs [22]. The third part of the study was a structured face-to-face interview survey questionnaire designed to gather information on areas of interest such as health, drug intake habits, and contact with drug treatment centers, identified in the qualitative part of the study. This article is based on results of the survey questionnaire.

### Study population

The investigation covers all five DCRs in Denmark. The five DCRs vary in setup and service. In the city of Odense, the DCR is part of a shelter and has a free needle exchange program. It is staffed by social workers, social educators, and nursing aides who provide health assistance. The service users have access to showers, laundry facilities, and free meals. The DCR in the city of Aarhus is also part of a shelter. It has an injection facility and a smoking facility reserved for smoking drugs such as heroin and cocaine, and a health clinic. Two DCRs in Copenhagen are part of a shelter, one of which operates 23 h a day and is integrated within a network of services such a health clinic and access to counselling. The facility has booths for injecting and a separate room for smoking. The other DCR is associated with a health clinic, but lacks a smoking facility. There is approximately a 30-min time limit to the length of stay in these two facilities. The third DCR in Copenhagen is a mobile DCR, which is staffed only by nurses, and has room for four service users at a time. There is unlimited and free access to drug paraphernalia in the Aarhus and Copenhagen DCRs. Paraphernalia is equipment such as syringes, needles, alcohol wipes, sterilized water, sets for cooking heroin, smoking foil, bicarbonate, smoking sets, and other tools used for drug intake. There is no limit to the length of stay in the mobile facility or the facilities in Odense and Aarhus. In 2014, there were 3564 unique service users registered at the five DCRs [8]. Our sample consisted of 154 service users of DCRs in Denmark. However, not all registered service users from 2014 were active service users at the time the survey was conducted in January 2015. Active service users are here defined as persons who are currently registered visitors of the DCR. This number varies as clients are sometimes absent for a period; some go to drug rehabilitation, some to jail, travel, or do not use the DCRs for a while and then they may appear again. For example, at one DCR (Aarhus), there were 205 registered service users since opening of the DCR, but only 35 were visiting the DCR at the time the survey was conducted. Because of this, it has not been possible to determine the precise size of the client pool nor was it possible to determine the degree to which our sample represents the population of service users of the DCRs.

### Survey instrument and administration

A survey was developed to be administered in the form of face-to-face interviews. As no Danish validated survey assessing health impact of DCRs on service users exists, this survey has been developed to fit the local Danish context. Expert panel validation was used and parties involved were the head physician of an addiction treatment center, the chiefs of staff and staff members from the DCRs, professional researchers experienced in survey design, a statistician, a researcher in the field of drug use, and associate professors involved in the study who have experience in approaching PWUD and engaging them in the study. Two pilot tests were conducted prior to the final survey. Each pilot test was followed by review in expert panel group sessions. The survey had 42 questions and took about 10–15 min to complete. It was administered on tablet computers using the Enalyzer Survey Solution System online [26].

All interviews were conducted between 5th January 2015 and 2nd February 2015. DCR staff was helpful in informing service users about the study and encouraging participation. Informational posters about the project were visible in the DCRs during the survey process. To gain heterogeneity, time of day and week varied to cover all possible opening hours. Some DCRs have extended opening hours following the so-called pay days, where people receive money (social benefits, retirement pension, etc.) due to extraordinarily high activity. Thus, interviews were conducted on weekdays and weekends, in the daytime and nighttime as well as on pay days and in between pay days. To avoid bias in regard to answering sensitive questions, no staff from the DCRs administered the questionnaires. The researchers undertook the study, in collaboration with nursing students who were carefully recruited and trained. The nursing students collectively interviewed 10 service users; the rest of the 154 interviews were conducted by the researchers. Convenience sampling was used. Eligible participants were current service users of the DCRs, age over 18 years, who smoke, sniff or inject drugs. Participants were required to have an adequate understanding of Danish in order to complete the interview survey without translation. Participants were recruited in the vicinity of the DCRs or inside the waiting areas and sampled consecutively. Total hours of data collection and total number of participants at each site were stratified and weighted according to the number of registered service users in the DCRs [27–29], to ensure adequate representation of possible subgroups. However, we had to spend additional time in the DCR with the fewest service users to recruit participants (Table 1).

**Table 1:** Data collection

	Registered service users at time of survey	Hours of survey data collection	Number of participants *n*
Aarhus	205	11	5
Odense	375	28	38
The mobile unit	270	8	8
Halmtorvet	1174	30	22
Skyen	2348	97	81

An interviewer approached the interviewee to describe the purpose of the survey and inform of the anonymity. Face-to-face interviews were conducted and answers were entered on computer tablets. Data was exported from Enalyzer to the statistical programs SPSS and R system for statistical analysis [30, 31]. Bivariate analysis was done for groups and subgroups of variables. Descriptive statistics were performed to summarize data. Categorical table analysis was performed to examine the associations between categorical exposures and outcome variables using Fisher’s exact test and chi-squared test.

Numbers of refusals to participate were 40. Out of respect for refusal to participate, no information on service users who refused were collected. In order to avoid double participation, service users were asked whether they had already been interviewed. A total of 146 of the 154 questionnaires were fully completed. Because some of the service users were recruited in the waiting area of the DCRs while they were in line to enter, eight of the questionnaires were not completed in full, as the service users were called in to the DCR before the survey interview was completed. Missing data were deleted in order to make a complete case analysis. Questionnaire domains were demographics, pattern of using DCRs, drug intake method, drugs used, drug rehabilitation, advice received at DCRs, overdose experience, contacts to health care, service users’ opinion about DCRs, and role of DCRs in rehabilitation. The STROBE Statement, whose aim is to strengthen the reporting of observational studies in epidemiology, was used to prepare this manuscript [32].

## Results

### Demographics of Danish DCR service users

To identify characteristics of this population, service users were asked about demographics, drug intake mode, disease status, and incarceration. As shown in Table 2, most participants of the survey were above 30 years of age—only 10 % were under the age of 30. Only 25 % of the participants were female, which is equivalent to the normal distribution ratio seen for gender in this population in Denmark [10]. Most participants had encountered drugs at a young age, and 60 % reported starting to use illicit drugs (like heroin and cocaine but not cannabis) before they were 19 years old. Danish citizens comprised 73 % of the participants, non-Danish EU citizens made up 8 %, and 18 % had non-EU citizenship. In the two smaller cities, Aarhus and Odense, all participants in the sample were Danish. A total of 70 % were single and 30 % cohabiting, and 45 % had no children. In response to the labor market attachment, participants were asked whether they were employed full-time or part-time, in job training, or students. These categories were grouped as working full or part time. Only 11 % of participants had full- or part-time work, 44 % were receiving social welfare payments, and 38 % were on early retirement. The group “other” covered no source of income or income by selling magazines specially provided for unemployed PWUD to sell. In regard to housing, the participants were asked whether they had stable or unstable housing, unstable housing being defined as being homeless, living in a shelter, on the street, or irregular sleeping accommodations with friends. In this sample, 60 % had stable housing.

Only 1 % inject subcutaneously, 63 % inject intravenously, 7 % sniff, and 37 % smoke. Of all participants who injected drugs, 87 % are Danish. Some participants reported more than one method of intake. A total of 80 % used the DCRs more than once a week and 42 % used DCRs more than five times a week. Furthermore, 71 % used the DCRs more than twice a day, and 22 % used it more than five times a day. Clearly, some participants were very frequent service users of the DCRs.

**Table 2:** Demographic characteristics and drug intake mode of participants of DCRs (*n* = 154)

	Total *n* (%) 154 (100)
Age, years	
20–29	15 (10)
30-39	47 (30)
40-49	55 (36)
≥50	37 (24)
Gender	
Male	116 (75)
Female	38 (25)
Age at first time of hard drug use, years^a^	
9–19	93 (60)
20–29	42 (27)
30–39	14 (9)
≥40	5 (3)
Nationality^a^	
Danish citizenship	113 (73)
EU citizenship other than Danish	13 (8)
Non-EU citizen	28 (18)
Residence status	
Single	108 (70)
Cohabiting	46 (30)
Parental status	
No children	66 (45)
Children	80 (55)
Work status	
Full time/part time work	16 (11)
Social welfare	65 (44)
Early retirement	56 (38)
Other	11 (7)
Housing status	
Stable	92 (60)
Unstable	62 (40)
Drug intake method	
Inject in a muscle or subcutaneous	1 (1)
Inject in a vein	96 (63)
Sniff	10 (7)
Smoke	56 (37)
Drugs used	
Cocaine	75 (49)
Heroin	62 (41)
Mix of heroin and cocaine	24 (16)
Methadone	24 (16)
Benzodiazepines	8 (5)
Ritaline	5 (3)
Amphetamines	3 (2)
Frequency of DCR use per week	
Less than 1 day a week	30 (20)
1 day a week	17 (11)
2–4 days a week	42 (27)
5–7 days a week	65 (42)
Frequency of use of DCR per day	
1 time per day	45 (29)
2–5 times per day	75 (49)
More than 5 times per day	33 (22)
Disease status	
Hepatitis C virus (HCV)	56 (39)
Hepatitis B virus (HBV)	13 (9)
HIV	5 (3)
Tuberculosis	5 (3)
None of these	80 (55)
Incarceration^b^	
Spent time in prison	118 (81)
Never been to prison	21 (14)
Prefer not to answer	7 (5)
Incarcerated 1–5 times	62 (53)
6–10 times	32 (28)
11–50 times	22 (19)
Use of health care system the past 3 months	
Health care clinic in or close to DCRs	44 (30)
Street nurse	9 (6)
General practitioner	44 (30)
Emergency room	24 (16)
Hospitalization	22 (14)
Outpatient contact	24 (16)
None of the above	55 (37)

^a^Total is 99 due to rounding error

^b^Only three (5 %) with HCV have never been in prison, Pearson chi-square *p* = 0.034. All participants with HIV have spent time in prison. *N* 71 (60 %) of those who spent time in prison, were age 9–19 when they started using illicit drugs

A total of 39 % report being infected with hepatitis C virus (HCV), 9 % with hepatitis B (HBV), 3 % with HIV, and 3 % with tuberculosis. There is a significantly higher prevalence of HCV among participants who injected drugs compared to those who smoke drugs (*p* = 0.000). Of the 96 persons who inject drugs, 48 persons (53 %) had HCV. Of the 55 persons who smoked, and answered questions on disease, only 9 persons or 17 % of the smoking group had HCV (Table 3). Of the 146 participants who completed the questionnaire in full, 81 % had been in prison, only 14 % answered that they had never been in prison and 5 % declined to respond (Table 2). Further cross analysis shows that 47 % had been in prison more than five times and 60 % of those who had been incarcerated were between the ages of 9–19 when they started using illicit drugs (Table 2). When examining the relationship between disease status and incarceration, only 5 % of those who have HCV had never been in prison (*p* = 0.034) and all persons who reported having HIV had spent time in prison (Table 2).

**Table 3:** Opioid substitution treatment (OST)

	*n* (%)^a^		*p*
Is or have been in OST			
	Yes	No	
Nationality^b^			0.016
Danish	102 (91)	10 (9)	
EU other than Danish	10 (83)	2 (17)	
Non-EU citizen	19 (70)	8 (30)	
Drug intake method^c^			0.002
Smoke	41 (75)	14 (25)	
Inject or sniff	90 (94)	6 (6)	
Drug intake method			
Nationality^d^	Smoke	Sniff or inject	0.000
Danish	29 (52)	83 (87)	
EU other than Danish	3 (5)	9 (9)	
Non-EU citizen	24 (43)	4 (4)	
Total	56 (100)	96 (100)	
Disease status^e^	Smoke	Inject	0.000
HCV	9 (17)	48 (53)	
HBV	2 (4)	11 (12)	
HIV	1 (2)	5 (6)	
Tuberculosis	4 (8)	2 (2)	
None of these	39 (75)	38 (42)	
Total	55 (100)	104 (100)	
Advised on OST			
	Yes	No	
Nationality (total)^f^			0.35
Danish	30 (67)	82 (77)	
EU other than Danish	11 (24)	16 (15)	
Non-EU citizen	4 (9)	8 (8)	
Total	45 (100)	106 (100)	
Nationality (advised OST)^g^	45 (30)	106 (70)	0.33
Danish	30 (27)	82 (73)	
EU other than Danish	11 (41)	16 (59)	
Non-EU citizen	4 (33)	8 (67)	
Nationality (smokers)^h^			1.0
Danish	11 (38)	18 (62)	
EU other than Danish	1 (33)	2 (67)	
Non-EU citizen	9 (39)	14 (61)	
Drug intake method^i^			0.13
Smoke	21 (38)	34 (62)	
Inject or sniff	24 (25)	72 (75)	
Is or have been in OST^j^			0.42
Yes	37 (28)	94 (72)	
No	8 (40)	12 (60)	

^a^The number in parentheses is the percentage of the noted subgroup

^b^Fisher’s exact test comparing nationality against have been in OST. Significantly more Danish have been in OST. Of the entire sample, only 30 % (*n* = 45) have been advised on OST

^c^Chi-squared test comparing drug intake method against have been in OST. Significantly more inject/sniff have been in OST

^d^Chi-squared test comparing nationality against drug intake method. Significantly more who inject/sniff are Danish, and significantly more who smoke are of other nationality than Danish

^e^Chi-squared test comparing disease status against drug intake method. Significantly more with HCV inject

^f^Chi-squared test comparing nationality within against advised on OST. Within the group who have been advised, a total of 67 % (*n* = 30) are Danish and a total of 33 % (*n* = 15) are of other nationality than Danish

^g^Fisher’s exact test comparing nationality against advised on OST. Within the advised Danish group 27 % (*n* = 30) of the Danes had been advised and 73 % (*n* = 82) of the Danes had not

^h^Chi-squared test comparing nationality of smokers against advised on OST

^i^Chi-squared test comparing drug intake method against advised on OST

^j^Chi-squared test comparing have been in OST against advised on OST

### Associations between nationality, OST, drug intake method, and response to staff advice on harm-reducing education

This study also examined the associations between nationality, type of drug used, and OST; in this Danish context, OST is defined as methadone, buprenorphine, or heroin-assisted treatment. Participants were asked whether they were currently in drug rehabilitation treatment like OST, had been in treatment or had never been in treatment. As shown in Table 3, a total of 91 % of participants with Danish nationality were or had been in OST treatment. Of non-EU participants, only 70 % were or had been in OST and 83 % of those with other EU-citizenship than Danish had been or were in OST. Hence, significantly more Danish citizens than both other EU and non-EU citizens were or had been participating in OST (*p* = 0.016). An analysis of participants who smoke drugs revealed that they make significantly less use of drug rehabilitation programs than participants who sniff or inject drugs (*p* = 0.002). While 25 % of the smoking group had never been in OST, only 6 % (*n* = 6) of those who injected had never been in OST (Table 3).

All participants were asked whether they had been advised by staff of the DCRs about how to enter drug rehabilitation like OST. Only 45 persons, which were 30 % of the total sample, reported having been advised of the opportunity to enter drug treatment program. Of participants who reported to having been advised on OST, 67 % (*n* = 30) were Danish citizens and 33 % (*n* = 15) were of other nationality than Danish (Table 3). Of the total Danish group (Table 3), 27 % (*n* = 30) had been advised on OST and 73 % (*n* = 82) had not. Of the total group whose nationality was EU other than Danish, 41 % (*n* = 11) had been advised on OST, and 59 % (*n* = 16) had not; of the non-EU citizens, 33 % (*n* = 4) had been advised on OST and 67 % (*n* = 8) had not. Among all people advised on OST, there is no nationality difference. Within drug intake method, only 38 % (*n* = 21) of those who smoked and 24 % (*n* = 25) of those who sniffed or injected reported having been advised on OST (Table 3). A further analysis of participants who smoke drugs showed significantly more (*p* = 0.000); in fact, 48 % (*n* = 25) of participants who smoke drugs were of other nationalities other than Danish, whereas only 13 % (*n* = 13) of those who injected drugs were of other nationalities other than Danish (Table 3). Further analysis on the group who smoke shows that when comparing against nationality, they are advised at an almost similar rate, Danish 38 %, EU other than Danish 33 %, and non-EU 39 % respectively (Table 3).

### Contacts to specialized health care and harm-reducing education

The study also examined contacts to specialized health care and how service users respond to staff advice on health-related issues and harm-reducing education in relation to safer drug use practices. Therefore, participants were asked which types of contact with health care providers the service user had had within the previous 3 months (Table 2) as well as if they have received treatment for disease within the past 2 years, which is the time the DCRs have existed at the time of the survey. Some of the participants had several contacts, and 37 % had no contact with these most common health care system facilities (Table 2). When asked whether participants had been advised to seek medical attention for signs of disease observed by staff, 39 % (*n* = 57) of participants responded positively. Of those advised to seek medical attention, 68 % had also been in treatment for disease within the past 2 years, which could indicate that participants who are advised to seek medical attention follow given advice as there was a significant relationship between being advised to seek medical attention and having been in treatment for disease (*p* = 0.003) (Table 4).

**Table 4:** Advice, education, and overdose

	*n* (%)		*p*
	Yes	No	
Treatment for disease			
Advised to seek medical help^a,b^			
Yes	39 (68)	18 (32)	0.003
No	37 (42)	52 (38)	
Was the education useful?			
Have been educated by staff in:			
Safe injection *n* 55 (100)	52 (95)	3 (5)	
Hygienic measures *n* 62 (100)	61 (98)	1 (2)	
Use DCR because of access to clean tools			
Educated in hygienic measures^c^			
Yes	55 (93)	4 (7)	0.024
No	25 (76)	8 (24)	
Become better at preventing overdose since using DCR			
Experienced overdose inside DCR^d^			
Yes	16 (80)	4 (20)	0.0042
No	54 (43)	75 (57)	
Experienced overdose outside DCR^e^			
Yes	44 (58)	32 (42)	0.018
No	28 (37)	47 (63)	

^a^ *N* 57 (which is 39 % of the total sample) had been advised on seeking medical help

^b^Chi-squared test comparing advised to seek medical help against have been in treatment. Significantly more who have been advised have also been in treatment

^c^Fisher’s exact test shows significantly more who have been educated in hygienic measures use DCR because of access to clean tools

^d^Chi-squared test shoving significant correlation between having experience of OD inside DCR to perceived ability to prevent an OD since starting to use the DCR

^e^Chi-squared test shoving significant correlation between having experience of OD inside DCR to perceived ability to prevent an OD since starting to use the DCR

As seen in Table 4, 57 % (*n* = 55) of the total number of participants who inject (*n* = 97*)* had previously been educated by staff at the DCR in safe injection practices, which are defined as follows: use of tourniquet, injecting in the right direction in relation to the heart, change of injection site, change of needle at each perforation of the skin, and use of vein scanning device. Among those who received education, a total of 95 % found it to be useful. Furthermore, 61 % (*n* = 62) of the participants who injected had been educated in hygienic measures, such as hand washing before injecting, the cleansing of injection site with an alcohol swab before injecting, and the use of sterile water for dissolving the drug. Out of those, 98 % found the education to be useful. Also, the service users who have been educated in hygiene reported that they use the DCRs because of access to clean tools to a higher degree than those that had not been educated (Table 5).

**Table 5:** Belief in becoming drug free and education on hygienic measures

Importance of DCR for becoming drug-free	Great importance	Some importance	Less importance	No importance	Total *n* (%)	*p*
Own belief in becoming drug free in the future^a^						0.052
Believe to great extent	27 (66)	10 (40)	7 (50)	25 (38)	69 (47)	
Believe to some extent	10 (24)	10 (40)	4 (29)	22 (33)	46 (31)	
Believe to lesser extent	3 (7)	5 (20)	3 (21)	10 (15)	21 (14)	
Believe to no extent	1 (2)	0 (0)	0 (0)	9 (14)	10 (8)	
Total *n* (%)	41 (100)	25 (100)	14 (100)	66 (100)	146 (100)	
Use the DCR because of access to clean tool *n* (%)						
	Yes	No	Don’t know			
Have been educated in hygiene^b^						0.01
Yes	55 (93)	4 (7)	0 (0)		59 (100)	
No	25 (71)	8 (23)	2 (6)		35 (100)	

^a^Monte Carlo chi-squared test comparing own belief in drug free future against importance of DCR for a drug-free future

^b^Fisher’s exact test showing significantly more who have been educated in hygiene use DCR because of access to clean tools

A total of 13 % (*n* = 20) of the sample had experienced an OD inside the DCR and 50 % (*n* = 76) had experienced an OD inside the DCR. Analysis shows there is a correlation between having experienced an OD either inside (Table 4, *p* = 0.0042) or outside (Table 4, *p* = 0.018) of a DCR and perceived ability to prevent an OD since starting to use the DCR (Table 4). Analysis on service users who inject showed that 54 % of service users reported becoming better at preventing OD since starting to use the DCR and that 55 % used the DCR to avoid dying from OD (Table 6).

**Table 6:** Perceptions and reasons for using DCR

*n* 147 (100)	Yes *n* (%)	No *n* (%)	Don’t know
Coming not to bother surroundings	105 (71)	36 (25)	6 (4)
Trust staff	129 (88)	11 (8)	7 (4)
Feel safe	131 (89)	15 (10)	1 (1)
Coming for access to clean tools	108 (74)	34 (23)	5 (3)
Come here to avoid OD^a^	55 (55)	41 (44)	1 (1)
Become better at preventing OD	52 (54)	45 (46)	

^a^Only injecting service users

This study also examined how important participants find the DCRs in terms of becoming drug free and the extent to which they believed in a drug-free future for themselves. Many service users reported a belief in a drug-free future and 90 % of those indicated that the DCRs was of great importance for becoming drug free. However, note that analysis indicated no significance in this outcome, although the *p* value was nearly significant (*p* = 0.052, Table 5), suggesting the value of further inquiry.

### Reasons for using the DCRs and perceptions of security

We next examined possible reasons among the service users for using the DCRs and service users’ perceptions of security in the DCRs. Therefore, participants of the survey were presented with questions regarding why they use the DCRs and how they feel about the DCRs. They were asked whether they use DCRs because of access to clean tools, avoiding public drug intake, whether they use DCRs to assist in preventing overdose, how important they found the DCRs in relation to becoming drug free in the future, and whether they trust staff and feel safe in the DCRs. There was a significant effect of having been educated in good hygiene on reporting use of DCRs because of access to clean equipment (*p* = 0.01, Table 5). Seventy-four percent of all the participants reported using the DCR because of access to clean tools. The participants were conscious that their public drug use bothers the people in the area and 71 % reported that one reason for using the DCR was to avoid bothering people in the neighborhood. In regard to the participants’ trust in staff and feeling safe, 88 % reported trusting staff and 89 % reported feeling safe in the DCRs (Table 6).

### Analyses by site

Finally, we compared the different DCRs to determine if there were differences in the population and services received (Table 7). In the DCRs in the two smaller cities of Odense and Aarhus, participants were exclusively Danish. In the Skyen facility in Copenhagen, where there is a busy smoking facility, there were the highest number of non-EU citizens and also the lowest number of female participants. However, only five participants were recruited in Aarhus, none of whom were female, which may not sufficiently represent the true gender ratio. The participants in Skyen also had a high frequency of DCR use per week as 53 % of participants use the DCR 5–7 days a week and 31 % of those use the DCR more than five times per day. In all the DCRs, there is a focus on medical advice; however, Skyen and Aarhus have the most PWUD who do not receive advice. Skyen is the place where the fewest (52 %) had been educated in safe injection and the only place where not all who had received this education found it useful. In all the other facilities, all the participants found education in safer injection practice and hygienic useful.

**Table 7:** Analysis by site

*n* (%)	Mobile, Copenhagen	Halmtorvet, Copenhagen	Skyen, Copenhagen	Odense	Aarhus
Nationality					
Danish	6 (75)	13 (59)	51 (63)	38 (100)	5 (100)
EU other than Danish	2 (25)	7 (32)	4 (5)	0 (0)	0 (0)
Non-EU citizen	0 (0)	2 (9)	26 (32)	0 (0)	0 (0)
Gender					
Male	4 (50)	16 (73)	66 (82)	25 (66)	5 (100)
Female	4 (50)	6 (27)	15 (18)	13 (34)	0 (0)
Frequency of DCR use per week					
Less than 1 day a week	3 (38)	5 (23)	8 (10)	14 (37)	0 (0)
1 day a week	3 (38)	2 (9)	7 (9)	4 (11)	1 (20)
2–4 days a week	0 (0)	7 (32)	23 (28)	9 (24)	3 (60)
5–7 days a week	2 (25)	8 (36)	43 (53)	11 (29)	1 (20)
Frequency of use of DCR per day					
1 time per day	4 (50)	5 (24)	20 (25)	15 (40)	1 (20)
2–5 times per day	4 (50)	10 (48)	36 (44)	21 (55)	4 (80)
More than 5 times per day	0 (0)	6 (28)	25 (31)	2 (5)	0 (0)
Advised to seek medical help					
Yes	7 (88)	11 (52)	25 (32)	14 (38)	1 (20)
No	1 (13)	10 (48)	52 (68)	23 (62)	4 (80)
Educated by staff in safe injection					
Yes	6 (75)	11 (58)	17 (52)	19 (56)	2 (67)
No	2 (25)	8 (42)	16 (49)	15 (44)	1 (33)
Was the education useful?					
Yes	6 (100)	11 (100)	14 (82)	19 (100)	2 (100)
No	0 (0)	0 (0)	3 (18)	0 (0)	0 (0)
Educated in hygienic measures					
Yes	7 (88)	10 (53)	18 (55)	24 (71)	3 (100)
No	1 (12)	9 (47)	15 (46)	10 (29)	0 (0)
Was the education useful?					
Yes	7 (100)	10 (100)	17 (94)	24 (100)	3 (100)
No	0 (0)	0 (0)	1 (6)	0 (0)	0 (0)
Smokers educated in better smoking practices					
Yes	0 (0)	1 (100)	21 (40)	0 (0)	0 (0)
No	1 (100)	0 (0)	31 (60)	0 (0)	0 (0)
Was the education useful?					
Yes		1 (100)	18 (86)		
No		0 (0)	2 (9)		
Don’t know			1 (5)		
Advised on OST					
Yes	3 (38)	7 (33)	32 (40)	2 (5)	1 (20)
No	5 (63)	14 (67)	48 (60)	35 (95)	4 (80)

## Discussion

The Danish DCRs appear to be working well in several respects; however, a subgroup of service users of nationalities other than Danish deserves more attention. Across sites, the non-Danish group had participated less in OST. While we thought it would be more difficult to make contact with the service users of non-Danish nationality, they were advised on OST at an almost similar rate. However, still 61 % of the non-EU citizens who smoke were not advised and significantly fewer who smoked had been in OST. Since the non-EU citizens may also be underrepresented in our study, this subgroup could be larger than the study shows. In the qualitative part of this national study, the DCR with a smoking section in Copenhagen is seen as the largest and busiest, with service users of many nationalities [22]. This study confirms the busy scene as Skyen has the highest percentage of PWUD who use the facility 5–7 days a week and up to 31 % who use the facility more than five times per day. Here, there were also the lowest percentage who received educational advice and the lowest percentage of participants who found the education useful (Table 7). The language barrier makes it difficult to reach non-Danish service users and build up relationships as many service users speak poor Danish and poor English. This makes it difficult to develop a therapeutic rapport with the service users. Therapeutic rapport is an approach known to be important to engage service users and bridge to specialized care and drug treatment [33], and in the qualitative part of this national study, we found that forging relationships is seen as essential for referrals to health care [22]. Unlike in the injecting room, the staff cannot remain present in the smoking room due to a negative health risk for the staff due to second-hand smoke exposure. In the injection room, there are better opportunities for longer conversations between the staff and service users and thereby to allow rapport to develop gradually. Conversations with those who smoke have to take place elsewhere, typically the entrance of the facility which does not provide space for intimate conversations. Therefore, it is no surprise that only 40 % of the participants in this facility who smoke drugs had been educated in better smoking practices. Nonetheless, 86 % of those who had been educated found it useful (Table 7). Service users who smoke may need more harm-reducing interventions, like advice on OST, safer drug use methods, and health care education, than this study reveals.

One intention of having DCRs is to provide a setting for bridge building and guiding the service users to more specialized health care when needed [8]. The current analysis shows that staff in DCRs recommended service users that they seek medical attention, and 68 % of those advised had also been in treatment for disease during the 2-year period the DCRs had existed at the time of the survey. The anonymous health care clinics located close to the DCRs are used by 30 % of the service users. Since DCRs are organized in such a way that staff in the DCRs cannot provide basic nursing interventions like wound care, these clinics may play a major role in trying to secure medical attention for these service users. The short physical distance between the locations of DCRs and health clinics could account for the high number of positive referrals seen in the sample. It would make sense to offer basic nursing interventions such as wound care at the mobile DCRs that are operated further away from health clinics. Offering this basic health care service in the context of a DCR should be considered.

Only 57 % of the participants reported having been educated in safe injection techniques and 60 % in hygienic matters. However, further questioning as to whether participants find the education from the staff on injection practice useful shows that up to 98 % of participants find it useful. We previously reported that staff are selective about time and place for education in safe injection practices and proper hygiene. It appears that the staff are successful at targeting service users who are able to apply the education [22]. This is similar to other studies indicating that DCRs are effective in facilitating educational advice regarding unsafe and unhygienic injection practice which contribute to less drug-related injury [15, 33, 34].

It is no surprise that only 30 % of participants had been advised on how to get into drug treatment, as many of the Danish service users in the DCRs were already in contact with or had been in drug treatment. This study shows that the Danish DCRs promote contacts to specialized health care and provide a place that offers many services that the service users find important in order to eventually become drug free. According to a report from 2015 on Danish DCRs, more than 2359 nursing interventions on health-related matters, more than 1295 referrals to health care offers, and 354 referrals to substitution treatment were registered as having taken place at DCRs in 2014. Our finding on positive referrals is supported by other studies on positive referrals to drug treatment from DCRs [22, 33]. The qualitative part of this national study [22] indicates that the relationship formed between service users and staff and the trust service users have in the staff may be an important element for bridge building to specialized health care. Using the DCRs turns a stressful situation into an un-stressful situation for the service users. Inside the safe setting of a DCR, there is an opportunity for clients to learn about harm reductive practices and drug treatment options. More research into the relationship building that takes place at DCRs could contribute with more information about how this work influences service users and their motivation and success as they transit into drug treatment. Finally, more research in the service users who smoke and are of other nationality than Danish could contribute to important knowledge of the background of these people and open possibilities for harm-reducing care for them.

### Limitations

There are several limitations to the study. The total service user pool was not possible to calculate due to discrepancy between registered number of DCR service users and service users active at the time of the survey. PWUD are a hidden population and the service users visiting the DCRs are anonymous. They register with an alias and some have several aliases. Some service users, often the ones who smoke or use cocaine, re-enter the DCRs more than 20 times a day. Therefore, the number of drug intake events does not correspond with the number of service users. Sometimes, service users who have been daily visitors for a period disappear. It is not known whether they are in treatment, hospitalized, incarcerated, or even dead but still they figure as registered clients. So the actual number of service users per month varies and is difficult to register. The statistical methods of the study are primarily bivariate analysis, determining simple descriptive associations.

It is also limitation that participants had to be able to speak Danish. Many DCR service users, especially those who smoke drugs, are of other nationalities other than Danish and are underrepresented in this study. Also, the study and survey were developed and embedded in a Danish context and therefore may not be readily generalized to populations of DCR service users in other countries.

## Conclusions

The study contributes information on self-reported drug intake behavior of DCR service users’ health and contact to the primary sector and drug treatment. In only 2 years, Denmark has succeeded in opening five DCRs and attracting PWUD with a prolonged chronic history. Danish DCRs provide a positive environment for staff to establish relationships with service users which includes offering advice and education on medical, technical, hygienic, and drug treatment issues as well as bridging access to specialized health care. At present, health providers in Copenhagen are challenged when communicating with DCR service users of other nationalities other than Danish as they have been less in OST and may not be advised at the same rate as the Danish service users. Also, service users who primarily smoke their drugs and are of other nationalities other than Danish are underserved; more research on clients who smoke their drugs is needed. Danish DCRs have been organized so that staff cannot provide medical attention within the DCR; therefore, anonymous health clinics located near the DCRs may play an important role in the health of the service users. When operating DCRs at a location further away from a health clinic, a larger body of nursing interventions such as wound care should be considered and possibly integrated into the current services.

## Additional files

Additional file 1: Survey Questionnaire Danish version. (PDF 130 kb)
Additional file 2: Survey Questionnaire English version. (PDF 203 kb)

## Abbreviations

DCR: Drug consumption room
PWUD: People who use drugs
OST: Opioid substitution treatment
