# Supplementary material for: A cross-sectional national survey assessing self-reported drug intake behavior, contact with the primary sector and drug treatment among service users of Danish drug consumption rooms
Source: Harm Reduct J. 2016 Oct 7;13:27. doi: 10.1186/s12954-016-0115-0 (PMC5055654; doi:10.1186/s12954-016-0115-0)
Supplement: Additional file 1: — Survey Questionnaire Danish version. (PDF 130 kb) [file 12954_2016_115_MOESM1_ESM.pdf]

Projekt Stofindtagelsesrum

Kære bruger af stofindtagelsesrummet

Vi kommer fra Institut for Sygepleje i København. Vi vil høre om du vil medvirke i en anonym undersøgelse af hvordan stofbrugere oplever stofindtagelsesrummene i Danmark. Det tager ca. 10-15 min. at besvare spørgeskemaet.

Med venlig hilsen projektgruppen: forskningsansvarlig Nanna Kappel, Jette Tegner og Eva Toth.

1. Dato (skriv som dag måned år, f.eks 010215)

❑ _ _ _ _ _ _

2. Hvor udfyldes spørgeskemaet? (Angiv kun ét svar)

Fixelance	Halmtorvet	Skyen	Odense	Århus
❑	❑	❑	❑	❑

3. Hvilket køn er du?
(Angiv kun ét svar)

Mand	Kvinde
❑	❑

4. Hvor gammel er du? Skriv et tal

_ _ _ _ _ _

5. Dit statsborgerskab (Angiv kun ét svar)

Dansk

❑

Andet:_ _

6. Hvilket land er din mor født i?(Angiv kun ét svar)

Danmark

❑

Andet:_ _

7. Hvilket land er din far født i?
(Angiv kun ét svar)

Danmark

❑

Andet

_ _

8. Hvilket land er du født i?
(Angiv kun ét svar)

Danmark

❑

Andet

_ _

9. Hvad er din samlivsstatus?
(Angiv gerne flere svar)

Gift	Er i forhold	Enlig	Enlig (separeret/skilt)	Enlig (enkestand)
❑	❑	❑	❑	❑

10. Hvor gammel var du, da du første gang begyndte at sniffe, ryge og injicere stoffer, som ikke var hash? (Angiv værdi)

❑ _ _ _ _ _ _

11. Hvordan er din boligsituation? (Angiv gerne flere svar)

Fast bolig	Skiftende bolig (f.eks. hjemløs, herberg, gaden, skiftende sovesteder hos venner)
❑	❑

12. Hvor lang tid har du brugt stofindtagelsesrummet? (Angiv gerne flere svar)

Mindre end ½ år	½-1 år	Mere end 1 år
❑	❑	❑

13. Hvor mange dage om ugen bruger du stofindtagelsesrummet? (Angiv kun ét svar)

Mindre end en dag	En dag	2 -4 dage	5-7 dage
❑	❑	❑	❑

14. Hvor mange gange indtager du typisk stoffer i stofindtagelsesrummet i løbet af en dag? (Angiv kun ét svar)

En gang	2-5 gange	Mere end 5 gange
❑	❑	❑

15. Hvor ofte har du taget stoffer udenfor stofindtagelsesrummet den sidste uge? (Angiv kun ét svar)

0-1 gang	2-5 gange	Mere end 5 gange
❑	❑	❑

16. Når du ikke bruger stofindtagelsesrummet til at tage stoffer, hvor gør du det så typisk? (Angiv kun ét svar)

Hjemme	Hos venner eller bekendte	Herberg/hotel	Offentligt (toilet/ trappeopgang/ park/på gaden o.l.)	Andet
❑	❑	❑	❑	❑

17. Hvilket stof har du taget / skal du tage ved dette besøg? (Angiv gerne flere svar)

Heroin	Kokain	Blanding af heroin og kokain	Amfetamin/ speed	Metadon	Ritalin	Sove-nervepiller såsom Flunipam, Nitrazepam, Diazepam eller Oxabenz	Andet
❑	❑	❑	❑	❑	❑	❑	❑

18. Hvordan tager du stoffet her på stedet? (sæt gerne flere krydser)
(Angiv kun et svar pr. spørgsmål)

	Ja	Nej
Injicerer i en muskel eller under huden	❑	❑
Injicerer i blodåre (kan trække blod)	❑	❑
Sniffer	❑	❑
Ryger	❑	❑

Følgende spørgsmål er kun til dig der fixer. Hvis du ikke fixer, spring over spørgsmål 19-22.

19. Er du nogensinde blevet vejledt af personale i stofindtagelsesrummet i sikker injektionsteknik (dette omfatter brug af stase, injektion i hjertets retning, skifte injektionssted, skift af nål ved hvert stik, veneskanner, m.m.)? (Angiv kun ét svar)

Ja	Nej	Ved ikke
❑	❑	❑

20. Var denne vejledning nyttig? (Angiv kun ét svar)

Ja	Nej	Ved ikke
❑	❑	❑

21. Er du nogensinde blevet vejledt af personale i stofindtagelsesrummet i god hygiejne ved injektion (dette omfatter vaske hænder før injektion, spritte hud af før injektion, brug af sterilt vand)? (Angiv kun ét svar)

Ja	Nej	Ved ikke
❑	❑	❑

22. Var denne vejledning nyttig?
(Angiv kun ét svar)

Ja	Nej	Ved ikke
❑	❑	❑

Følgende spørgsmål er kun til dig der ryger. Hvis du ikke ryger, spring over spørgsmål 23 og 24.

23. Er du nogensinde blevet vejledt af personale her i bedre rygevaner (ikke dele pibe, ikke ryge aske, skifte filtre, bruge mundstykke for at beskytte læber, bruge læbepomade, tandbørstning, rygefolie, undgå brug af salmiakspiritus, brug af bikarbonat)?
(Angiv kun ét svar)

Ja	Nej	Ved ikke
❑	❑	❑

24. Var denne vejledning nyttig?
(Angiv kun ét svar)

Ja	Nej	Ved ikke
❑	❑	❑

25. Når du tager stoffer udenfor rummet, har du så nogensinde oplevet overdosis?
(Angiv kun ét svar)

Ja	Nej
❑	❑

26. Når du tager stoffer indenfor rummet, har du så nogensinde oplevet overdosis?
(Angiv kun ét svar)

Ja	Nej
❑	❑

27. Er du blevet bedre til at forebygge overdosis for dig selv eller andre efter du er begyndt at benytte stofindtagelsesrummet?

(Angiv kun ét svar)

Ja	Nej
❑	❑

28. Er du, eller har du været i behandling for stofafhængighed (tæl både metadonbehandling, heroinbehandling, suboxone behandling med)? (Sæt gerne flere krydser)

Jeg er i behandling	Jeg har tidligere været i behandling	Jeg har aldrig været i behandling
❑	❑	❑

29. Har du fået rådgivning i stofindtagelsesrummet om, hvordan du kan komme i misbrugsbehandling?
(Angiv kun ét svar)

Ja	Nej	Ved ikke
❑	❑	❑

30. Er du blevet vejledt af personale i stofindtagelsesrummet til at søge behandling for sygdom?

(Angiv kun ét svar)

Ja	Nej	Ved ikke
❑	❑	❑

31. Er du blevet mere opmærksom på tegn på sygdom efter du er begyndt at benytte stofindtagelsesrummet?
(Angiv kun ét svar)

Ja	Nej	Ved ikke
❑	❑	❑

32. Har du været i behandling for sygdom indenfor de sidste to år? (Angiv kun ét svar)

Ja	Nej	Ved ikke
❑	❑	❑

33. Hvordan er din tilknytning til arbejdsmarkedet?
(Angiv gerne flere svar)

Fuldtids arbejde	Deltids arbejde/ flexjob/ jobprøvning	Kontanthjælp	Førtids pension	Studerende	Anden lønindtægt, også Hus Forbi/ Illegal	Syge dagpenge	Andet
❑	❑	❑	❑	❑	❑	❑	❑

34. Har du indenfor de sidste 3 måneder været i kontakt med? (Angiv gerne flere svar)

(Angiv gerne flere svar)

Sundhedsrum	Kontakt med gadesygeplejerske	Egen læge	Skadestue	Indlagt på hospital	Ambulatorium (f.eks. hiv eller hepatitis behandling)	Ingen af de nævnte
❑	❑	❑	❑	❑	❑	❑

35. Hvad er din opfattelse af stofindtagelsesrummet?

(Angiv kun et svar pr. spørgsmål)

	Ja	Nej	Ved ikke
Er åbningstiden passende?	❑	❑	❑
Er tiden du må være i stofindtagelses-rummet passende?	❑	❑	❑
Er reglerne i stofindtagelses-rummet retfærdige (ingen handel, ikke assistere andre, man skal være over 18 år, og alm hensyn til hinanden)?	❑	❑	❑
Er sanktioner i stofindtagelses-rummet retfærdige (f.eks. karantæne, midlertidig bortvisning)?	❑	❑	❑
Har du tillid til personalet?	❑	❑	❑
Føler du dig tryg i stofindtagelses-rummet?	❑	❑	❑
Kommer du her for ikke at genere folk i nærmiljø/naboer med dit stofbrug?	❑	❑	❑
Kommer du her på grund af rent værktøj og hygiejne?	❑	❑	❑
Kommer du her for ikke at risikere at dø af overdosis?	❑	❑	❑

36. I hvor høj grad har du tiltro til, at du kommer ud af dit illegale stofbrug?

(Angiv kun ét svar)

I høj grad	I nogen grad	I mindre grad	Slet ikke
❑	❑	❑	❑

37. Siden du er begyndt at komme i stofindtagelsesrummet, har du oplevet en forbedring af din helbredstilstand?

(Angiv kun ét svar)

Ja	Nej	Ved ikke
❑	❑	❑

38. Hvilken betydning har det for dig at komme i stofindtagelsesrummet i forhold til følgende (Angiv kun et svar pr. linje)?

(Angiv kun et svar pr. spørgsmål)

	Stor betydning	Nogen betydning	Mindre betydning	Ingen betydning
I forhold til injektionsteknik	❑	❑	❑	❑
I forhold til rygeteknik	❑	❑	❑	❑
I forhold til sundhedstilstand	❑	❑	❑	❑
I forhold til kontakt til misbrugsbehandling	❑	❑	❑	❑
I forhold til fremtidig stoffrihed	❑	❑	❑	❑

39. Sæt kryds ved det der passer på dig i forhold til at have børn

(Angiv gerne flere svar)

Har ingen børn	Har børn under 18 år	Har børn over 18 år	Bor sammen med mindst et af dine børn	Børn bor sammen med den anden forælder	Børn er flyttet hjemmefra	Børn er anbragt i pleje	Ønsker ikke at svare
❑	❑	❑	❑	❑	❑	❑	❑

40. Har du været dømt og/eller afsonet fængselsstraf?

(Angiv kun ét svar)

Ja	Nej	Ønsker ikke at svare
❑	❑	❑

41. Hvor mange gange har du været dømt og/eller afsonet fængselsstraf? Skriv et tal

(Angiv værdi)

❑ _ _ _ _ _ _

42. Er du eller har du været smittet med nogle af følgende sygdomme? (Angiv gerne flere svar)

(Angiv gerne flere svar)

Hepatitis B	Hepatitis C	HIV	Tuberkulose	Ingen af de nævnte
❑	❑	❑	❑	❑

Tusind tak for din deltagelse
