# Supplementary material for: A cross-sectional national survey assessing self-reported drug intake behavior, contact with the primary sector and drug treatment among service users of Danish drug consumption rooms
Source: Harm Reduct J. 2016 Oct 7;13:27. doi: 10.1186/s12954-016-0115-0 (PMC5055654; doi:10.1186/s12954-016-0115-0)
Supplement: Additional file 2: — Survey Questionnaire English version. (PDF 203 kb) [file 12954_2016_115_MOESM2_ESM.pdf]

Projekt Drug Consumption Room

Dear service user of the drug consumption room

We are from Metropolitan University College, Department of Nursing in Copenhagen. We would like to invite you to participate in an anonymous survey regarding the drug Consumption room. It takes about 10-15 minutes to complete the survey.
Kind regards the project group: Nanna Kappel, Jette Tegner og Eva Toth.

1. Date

❑ _ _ _ _ _ _

2. Which DCR

Fixelance	Halmtorvet	Skyen	Odense	Aarhus
❑	❑	❑	❑	❑

3. Gender

Man	Woman
❑	❑

4. Age

_ _ _ _ _ _

5. Citizenship

Danish

❑

Other:_ _

6. In which country is your mother born?

Denmark

❑

Other:_ _

7. In which country is your father born?

Denmark

❑

Other

_ _

8. in which country are you born?

Denmark

❑

Other

_ _

9. Civil status

Married	In a relationship	Single	single (separated/divorce)	Single(widowed)
❑	❑	❑	❑	❑

10. How old were you the first time you started using illicit drugs (like heroin or cocaine but not including cannabis)?

❑ _ _ _ _ _ _

11. Current living circumstances

Stable living	Unstable living (homeless, shelter, street, changing places with friends)
❑	❑

12. How long have you used the drug consumption room?

Less than ½ year	½-1 year	More than 1 year
❑	❑	❑

13. How many days a week do you use the drug consumption room?

Less than one day	One day	2 -4 days	5-7 days
❑	❑	❑	❑

14. How many times per day do you typically use drugs in the drug consumption room?

Once	2-5 times	More than 5 times
❑	❑	❑

15. How often have you taken drugs outside the drug consumption room the past week?

0-1 time	2-5 times	More than 5 times
❑	❑	❑

16. When you do not use the drug consumption room to consume drugs, where do you typically do it?

Home	Friend’s house	Shelter/hotel	Public (toilet/ stairway/ park/street)	Other
❑	❑	❑	❑	❑

17.Which drug do you consume on this visit?

Heroin	Cocaine	Mix heroin and cocaine	Amphetami ne/ speed	Methadone	Ritalin	Sleeping pills/ benzodiaze pines	Other
❑	❑	❑	❑	❑	❑	❑	❑

18. How do you take the drug here?

	Yes	No
Inject in a muscle or under skin	❑	❑
Inject in a vein	❑	❑
Sniff	❑	❑
Smoke	❑	❑

The following questions are for you who inject. If you do not inject skip questions 19-22.

19. Have you ever been educated by staff of the drug consumption room in safe injection practice (use of tourniquet, injection direction, change of injection site, change of needle, vein scan)?

Yes	No	Don't know
❑	❑	❑

20. Was this education useful?

Yes	No	Don't know
❑	❑	❑

21. Have you ever been educated by staff of the drug consumption room in hygienic measures (hand washing, alcohol swipe before injection, use of sterile water)?

Yes	No	Don't know
❑	❑	❑

22. Was this education useful?

Yes	No	Don't know
❑	❑	❑

The following questions are for you who smoke. If you do not smoke skip questions 23 and 24.

23. Have you ever been educated by staff of the drug consumption room in safer smoking practices (not sharing pipe, not smoking ashes, change of filters, use of mouthpiece, chap stick, toothbrushing, smoking foil, avoid ammonium hydroxide, use of bicarbonate)?

Yes	No	Don't know
❑	❑	❑

24. Was this education useful?

Yes	No	Don't know
❑	❑	❑

25. When you take drugs outside the drug consumption room, have you ever had an overdose?

Yes	No
❑	❑

26. When you take drugs inside the drug consumption room, have you ever had an overdose?

Yes	No
❑	❑

27. Have you become better at preventing overdose since starting to use the DCR?

Yes	No
❑	❑

28. Are you or have you been in drug treatment like opioid substitution treatment (metadone treatment, buprenorphine, heroin assisted treatment)?

I am in treatment	I have previously been in treatment	I have never been in treatment
❑	❑	❑

29. Have you been advised by staff on how to enter a drug treatment program like opioid substitution treatment?

Yes	No	Don’t know
❑	❑	❑

30. Have you been advised by staff to seek treatment for disease?

Yes	No	Don’t know
❑	❑	❑

31. Have you become more aware of signs of disease since starting to use the drug consumption room?

Yes	No	Don´t know
❑	❑	❑

32. Have you been in treatment for disease within the past two years?

Yes	No	Don’t know
❑	❑	❑

33. How is your employment status?

Full time work	Part time work	Unimployment	Early retirement	Student	Selling magazines like Homeless/ Illegal	Sickness wage	Other
❑	❑	❑	❑	❑	❑	❑	❑

34. Have you within the past 3 months been in contact with?

Health care clinic	Street nurse	General practitioner	Emergency room	Hospitalization	Outpatient contact	None of these
❑	❑	❑	❑	❑	❑	❑

35. What is your perception of the drug consumption room?

	Yes	No	Don’t know
Are the opening hours appropriate?	❑	❑	❑
Is the time you can stay appropriate?	❑	❑	❑
Are the rules fair (no dealing, no assisting each other, age above 18, courtesy of others)?	❑	❑	❑
Are the sanctions fair (temporary exclusion)?	❑	❑	❑
Do you trust staff?	❑	❑	❑
Do you feel safe in here?	❑	❑	❑
Use drug consumption room to avoid public drug intake?	❑	❑	❑
Use drug consumption room because of access to clean tools and hygiene?	❑	❑	❑
Use drug consumption room to avoid overdose?	❑	❑	❑

36. To which extent do you believe in a drug free future for yourself?

To great extent	To some extent	To lesser extent	To no extent
❑	❑	❑	❑

37. Since you started using the drug consumption room, have you experienced an improvement in your health?

Yes	No	Don’t know
❑	❑	❑

38. What importance does coming in the drug consumption room have for you in regard to the following?

	Great importance	Som importance	Less importance	No importance
In relation to injection technique	❑	❑	❑	❑
In relation to smoking practices	❑	❑	❑	❑
In relation to health	❑	❑	❑	❑
In relation to contact to drug treatment	❑	❑	❑	❑
Importance of DCR for becoming drug-free	❑	❑	❑	❑

39. Mark what is true for you in relation to children

Have no children	Have children under 18 years	Have children above 18 years.	Living with at least one of my children	Children living with other parent	Children moved away	Children in foster care	Do not wish to answer
❑	❑	❑	❑	❑	❑	❑	❑

40. Have you been convicted or incarcerated?

Yes	No	Do not wish to answer
❑	❑	❑

41. How many times have you been convicted or incarcerated?

❑ _ _ _ _ _ _

42. Are you or have you been infected with some of the following?

Hepatitis B	Hepatitis C	HIV	Tuberkulosis	None of the mentioned
❑	❑	❑	❑	❑

Thank you for your participation
